# Organ Transplantation in Iran; Current State and Challenges with a View on Ethical Consideration

**DOI:** 10.3390/jcm7030045

**Published:** 2018-03-05

**Authors:** Mehrzad Kiani, Mahmoud Abbasi, Mehdi Ahmadi, Bahare Salehi

**Affiliations:** 1 Department of Medical Ethics, Faculty of Traditional Medicine, Shahid Beheshti University of Medical Sciences, Tehran 1985717443, Iran; mehrkia55@gmail.com; 2 Medical Ethics and Law Research Center, Shahid Beheshti University of Medical Sciences, Tehran 1985717443, Iran; dr.abbasi@sbmu.ac.ir; 3 Razi Vaccine and Serum Research Institute (RVSRI), Agricultural Research, Education and Extension Organization (AREEO), Karaj 3197619751, Iran; m.ahmadi1981@gmail.com

**Keywords:** ethical issues, organ transplantation, organ donation, brain death, extension of organ transplantation

## Abstract

Organ transplantation is a new issue in medical science. It is an important achievement and a sign of the progression and ability of medical centers around the world. Governments, populations, the medical community and people involved in culture, art, and media all have a decisive role in the culture of organ donation, which is the only way to guarantee that the healthy organs of a brain-dead person can continue to work and save the lives of people in need of organ transplantation. The brain death phenomenon and its possible application in organ transplantation, while offering new hope for the salvation of a number of patients, has led to many ethical, cultural, and legal issues. Ethical issues in organ transplantation are very complicated due to many social factors such as religion, culture, and traditions of the affected communities. The ethical and legal points of removing organs from the body of a living or cadaveric source, the definition of brain death, the moral and legal conditions of the donor and the recipient, and the financial relationship between them and many others, are all critical issues in organ transplantation. While there may be no available explicit solution to these issues, they should be rigorously considered by the experts. Efforts to systematically eliminate barriers and solve problems in organ transplantation, can not only reduce the costs of maintaining brain-dead patients and encourage patients that need organ transplantation but can also prevent immoral and illegal activities. In this paper, we have reviewed the most important and current challenges in organ transplantation with a view to the ethical considerations, and we have suggested some strategies to extend it in Iran.

## 1. Introduction

Around the world, organ transplantation has been conducted successfully for over 60 years. In Iran, good progress in organ transplantation has been made in recent decades, including organ transplantation from a living or cadaveric source and the presence of transplant centers. On the other hand, the high and rising incidence of chronic diseases such as hypertension, diabetes, obesity, and kidney disease will greatly increase the need for organ transplants in Iran in the coming years, so coherent and targeted plans should be prepared for the future. Many of these chronic diseases ultimately lead to organ failure (of the kidney, liver, heart, and so forth) which results in physical disability or death [1]. Organ transplantation is the best cure for these complications [2,3].

Brain death is defined as the irretrievable loss of all functions of the brain, including the brainstem [4,5,6]. Other organs such as the heart, liver and kidneys still work after brain death, but they only continue to function for a short duration. Although modern ICU (intensive care unit) technology can somewhat help to keep the other organs for longer, there is only one way to extend the life of the heart, kidneys, lungs, and other organs of the brain-dead patients: that is organ transplantation from the individual to the patient who needs the organ.

The ability to transplant organs has been a long-term wish of mankind throughout history, and today the medical world has reached this ambition [7]. Organ transplantation has existed from the beginning of the Chinese, Indian, Egyptian, Babylonian, and Islamic epochs. From the earliest successful human transplant by Eduard Zirm in 1905 [8], to date, with the advancement of medical knowledge, oocyte cells, the heart, kidneys, lungs, and most tissues of the body have been exchanged and transplanted. The timeline of first successful transplants in Iran is shown in Table 1.

Organ transplantation from a living person and brain-dead patients in Iran, while not having a long history, is an effective way to treat refractory diseases, enabled by rapid advances in medical science. Living donors can be used for kidney transplantation. However, for the heart, lung, and liver, and for corneal tissue, it is necessary to transplant the organ/tissue from the cadaver. Considering that, today, the advancement of knowledge and technology in the field of organ and tissue transplantation is very significant [9], and that the ethical issues on organ transplants have also changed remarkably [10,11,12], new topics concerning transplantation have attracted the attention of lawyers, religious leaders and moral philosophers; different cultures and religions in societies also have a great influence on ethical decision making and the transplantation of organs [13,14].

Organ transplantation in Iran has a history of over forty years [11], and since many years ago the concept of organ transplantation was established by the adoption of the law of organ donation. Many attempts have been made to construct this humanitarian action with ethical considerations, but the organ donation rate relative to the number of the brain-dead people is very low. Base on Iranian Society of Organ Donation report in 2017, 5 to 8 thousand potential brain deaths/year. The possible organ donor number is 2500 to 4000 and actual donation rates is 808. I addition they have reported that mean family consent rate in the country is 70% and transplant list patients are more than 25,000 [15].

The lack of organ donation culture, the late diagnosis of brain death, and the lack of specialized facilities or well-equipped hospitals with organ transplantation capabilities are challenges in this area. In the future, organ transplantation in Iran appears to face several challenges. In this review article, we have tried to study the most important ethical considerations and challenges facing organ transplantation and its developmental strategies in Iran.

## 2. Brain Function in Coma, Vegetative State, Brain Death and Related Disorders

A true and reliable assessment of the arousal and awareness of consciousness in patients with severe brain damage is very important for their management [16]. Advancement in intensive care has augmented the number of patients who stay alive with severe acute brain damage [16].

**Figure 1:**
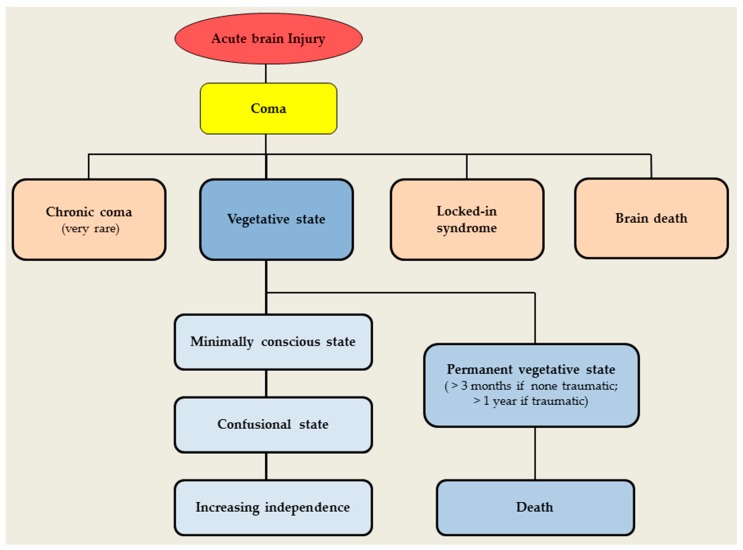
The Flowchart of cerebral insult, coma, vegetative state, brain death, and related disorders [17].

Even though the majority of patients recover from their coma within the first few days after the damage, others will take more time and go through different stages such as coma, chronic coma (very rare), locked-in syndrome, vegetative state (minimally conscious state, permanent vegetative state), and brain death (Figure 1).

A coma is categorized by the absence of arousal and, thus, also of consciousness: coma is a state of unresponsiveness in which the patient lies with the eyes closed, cannot be aroused, and has no awareness of themselves or their surroundings [17]. Stimulation will not result in spontaneous periods of wakefulness and eye-opening in patients in a coma, which differs from patients in a vegetative state [18].

Comas can result from diffuse bihemispheric cortical or white-matter injury after neuronal or axonal damage, or from focal brainstem lesions that affect the pontomesencephalic tegmentum or paramedian thalami bilaterally [17].

Patients in a vegetative state are awake but are unaware of themselves or their surrounding [19]. A persistent vegetative state is defined as a vegetative state remaining for one month or more after an acute traumatic or non-traumatic brain injury and it does not infer irreversibility [20]. A permanent vegetative state is irreversible. In vegetative state patients, the brainstem is generally spared, whereas the grey and white matters of both cerebral hemispheres are extensively and severely damaged. Overall, the cortical metabolism of patients in a vegetative state is 40–50% of the normal range of values [21,22,23,24,25,26,27]. In a permanent vegetative state (such as 3 months after non-traumatic or one year after traumatic brain damage), brain metabolism values drop to 30–40% of the normal range of values [21].

An apallic state or syndrome is an archaic term for a situation that is now deemed equivalent to a vegetative state. The term neocortical death has been used contrarily by different authors. Some refer to it as a vegetative state with the absence or substantial slowing of electrocortical activity as measured by an electroencephalography (EEG), similar to the characteristics of the vegetative state. Other authors equate neocortical death with the ostensible death of all neurons of the cerebral cortex. It’s not entirely clear whether this term denotes a clinical syndrome or its electrical, anatomical, or pathologic features [20].

The concept of brain death as the death of the patient is largely accepted [28]. Most countries have provided recommendations for the diagnosis of brain death, but the diagnostic criteria vary from country to country [29]. Some depend on the death of the brainstem only [30], others require the death of the entire brain comprising the brainstem [31]. Nevertheless, the clinical assessments for brain death are similar: requiring the loss of all brainstem reflexes and the demonstration of continuing apnoea in a persistently comatose patient [32]. Functional imaging with cerebral perfusion tracers and single photon emission CT [33,34,35,36] or cerebral metabolism tracers and PET [37] usually show a “hollow skull phenomenon” in patients who are brain dead, confirming the complete absence of neuronal function in the brain.

Miller et al. [38] studied brain death in Islam. They illustrated that brain death has been recognized as representing true death by many medical organizations and Muslim scholars, comprising the Islamic Fiqh Academies of the Organization of the Islamic Conference and the Muslim World League, the Islamic Medical Association of North America, and other faith-based medical organizations as well as legal decisions by multiple Islamic nations. Nevertheless, consensus in the Muslim world is not unanimous, and a substantial minority accepts death by cardiopulmonary standards only. Truog and Miller [39] seek to change the conversation about brain death by highlighting the distinction between brain death as a biological concept versus brain death as a legal status. They stated that brain death does not agree with any biologically credible description of death has been known for decades. Despite that, this fact has not threatened the acceptance of brain death as a legal status that authorizes individuals to be treated as if they are dead. The similarities between “legally dead” and “legally blind” reveal how we may legitimately select bright-line legal descriptions that do not cohere with biological reality. Not only does this contradistinction bring conceptual coherence to the conversation about brain death, but also it has practical implications as well. Also, they illustrated that formerly brain death is recognized as a social construction not grounded in biological reality, they make the probability of changing the social construction in ways that may better support both organ donors and recipients alike.

We emphasize that the discussion of states of impaired consciousness other than brain death is solely for the purpose of differential diagnosis. Death determination and organ donation pertains only to brain death and not to the related states.

The characteristics of a patient in different situations after acute brain injury including coma, vegetative state, brain death and related disorders are summarized in Table 2. These characteristics are not evident in every patient.

## 3. Overview of Organ Transplantation from a Living Donor and a Brain-Death Donor

A living-donor transplant is a surgical process which removes an organ or tissue from a living individual and places it in another individual whose organ is no longer functioning accurately. The reputation of living-organ donation has increased noticeably in recent years as a substitute to deceased-organ donation as result of the rising need for organs for transplantation and the shortage of accessible deceased-donor organs. Individuals can donate one of their two kidneys, and the remaining kidney is able to perform the vital functions. The most common type of living-donor procedure is living-kidney donation. Additionally, living donors can donate a portion of their liver and the remaining liver will regenerate, growing back to almost its original size, while functioning normally. Living-donor organ procedures are not limited to the kidney and liver (the most common types of living-donor organ transplantation) and living people may also donate tissues such as blood, skin and bone marrow for transplantation.

Brain death has distinctive implications for organ donations that it is a potential therapy for recipients [40]. Brain death is determined in the hospital by one or more physicians privileged to make brain death determinations and they not associated with the transplantation team [4]. The various reasons for brain death comprise cerebrovascular injury (for example, a stroke or aneurysm), trauma to the brain (such as a severe head injury caused by a motor vehicle crash, gunshot wound, fall or blow to the head), anoxia (such as in a drowning or heart attack where the patient is revived, but not before a lack of blood flow/oxygen to the brain has caused brain death) and brain tumors. The aforementioned reasons are some of the main causes, but there are other causes of brain death.

## 4. Risks Associated with Living-Organ Donation

The surgical process of transplanting an organ from a living-organ donor has become a fairly standard procedure, but it carries a level of risk for the donor equal to any other main surgery. The risks associated with living-organ donation involve short and long-term health risks associated with the surgical process, organ function and psychological problems subsequent to organ donation. The general surgery-related risks of organ donation include pain, blood loss, infection at the incision site, the potential need for blood transfusions, incisional hernia, medication side effects, pneumonia, abdominal hernia, blood clots, and, in rare cases, death. Commonly, for organ recipients, the risk of transplantation surgery is low as it is a potentially lifesaving process. Nevertheless, donating an organ can expose a healthy individual to the risk of and recovery from redundant major surgery.

Long-term follow-up information on living-organ donors is restricted, and studies are partial. In general, accessible information shows that organ donors fare very well over the long term. The follow up of short and long-term risks after living donation is important to confidently support informed consent and to provide public trust in living donation [41].

Donating an organ may also lead to negative psychological problems after donation. The donor and/or recipient may face surgical complications. The transplanted organ may not work right away. There is also the chance it will not work at all. Donors may feel anxious, sad, angry, or resentful after surgery. It is important that the living donors must be made aware of the physical and psychological risks involved before they consent to donating an organ. According to the type of donation, the known health risks associated with living-organ donation vary. To decrease risks, the donor and recipient need to have extensive testing to certify their eligibility to donate.

## 5. Golden Time in Brain Death for Organ Transplantation

At a global level, the organs of nearly 50% of brain-dead patients are being donated [42]. However, the organ donation of brain-dead patients is novel in Iran, thus the rate of organ donation of brain-dead patients can be very encouraging. More than one million people in the world currently benefit from organ transplant therapy, and many of these patients have survived for more than 25 years [2]. The important point in transplanting brain-dead patients is the golden time for organ transplantation. The golden time for organ transplantation is only a few hours to a few days—the specific duration varies for different organs. The process in Iran is made more difficult by the need to obtain donation consent from family members of a brain-dead patient who does not have an organ donor card. In many cases, families do not easily consent to organ donation, and the process of satisfying their concerns can sometimes take up to four days, during which, the golden time disappears for the donation of some vital organs, including the lungs. However, organ transplantation is the only chance for survival in patients with advanced heart and liver failure and the like. Currently, numerous organ transplantation centers are active in Iran, such as centers for the heart, kidney, liver, bone marrow, lung, and other organs/tissues.

## 6. Brain Death and Organ Transplantation Culture in Iran

Despite the passage of several years since the enactment of the organ transplantation law in Iran, a culture of organ transplantation has not yet been institutionalized in the country due to numerous problems, and, despite the high scientific and specialized ability to carry out transplantations, the process still encounters many problems. Statistics show that between 7 and 10 patients in need of an organ transplant will die due to not receiving the suitable organ [15]. Furthermore, there is low donation culture among the family of patients with brain death. This situation causes some people in need of transplantation to inevitably return to the black market for the purchase of organs: a market that confuses them and causes more problems [43].

Experts believe that low statistics are not due to the lack of facilities or technology, nor are they due to lack of a sacrificial spirit in the general population. The main reason is a weakness of organ transplantation culture in Iran: the issue of brain death must be properly explained to the community and the prevailing culture should be prepared in to enable people to express their altruism in critical situations and make a reasonable decision. According to experts in this field, a poor culture and a lack of adequate knowledge in the area of organ transplantation is one of the main obstacles to the progression of organ transplantation in developing countries such as Iran [44,45,46]. Currently, there is no comprehensive system in all hospitals in Iran, to coordinate the quick release of information on the existence of a patient with brain death. This problem is also more pressing in the hospitals in smaller cities and provinces. The lack of proper care for the brain-dead patient at the source hospital (necessary for keeping vital organs healthy for donation) is among the problems occurring in many unequipped hospitals. Poor legal protection to support the donor families is another problem that was pointed out by the experts. In most countries, the cost of treatment for brain death is paid by insurers [47], but insurers are not obliged to do so in Iran, although insurance does not provide special protection in support of patients who have received new organs; most of the costs of organ transplants are paid by the recipient of the organ transplant which is considered to be one of the most expensive surgeries.

## 7. Challenges Related to Ethical Considerations in Organ Transplantation

The tremendous advances in medical science and surgical techniques over the past few decades have saved many lives and have broadly improved the quality of life for patients. Nowadays, people with chronic illness get rid of the devastating consequences of these illnesses with the help of well-trained professionals using new drugs and careful treatment programs. However, the existence of human mistakes in today’s complex, technological conditions has doubled the need for considering the ethical issues in the medical field.

Ethical issues, especially those raised in areas such as medicine, are often global and transnational; as the achievements of medical science and research are global and inclusive, the discussion on these issues, therefore, requires a global, extra-religious, and transcultural approach [48,49].

Organ transplantation is one of the important topics in the field of medical ethics, which is nowadays commonplace in many countries [50]. The ethical issues in organ transplantation are very complicated due to various factors [51,52]. Religion, culture, and traditions in society are among these factors, which should be seen alongside the scientific discussion of medical ethics and its legal discussion [53]. Ethical and legal points in removing an organ from a relative or non-relative living person, organ donation consent and how to get it, justice and resource allocation, the primary rights of donors and recipients, observance of ethical standards in the processing of the organs of brain-dead patients, time of brain deaths and mandate issues are all just a gateway to the ethical issues in organ transplantation. Furthermore, research and the advent of new transplant technologies also attracts attention to the use of these technologies and the ethical issues surrounding them. Since ethical discussions and criticisms should always be of concern to researchers and authorities, it is necessary to look seriously at the various issues in transplantation and, with the advent of new phenomena and sciences related to this field, we intend to provide logical solutions in accordance with the culture, beliefs and laws of Iran. Since the Islamic Republic of Iran, either in the field of Islam or medicine, is one of the important and influential countries in the Islamic world and especially in the region, the need for a comprehensive review of the ethical considerations in Iran surrounding organ transplantation and the use of new technologies, especially the research and technological facilities of modern medical science, is necessary. This review will also help to explain the ethical codes and clarify some legal issues, as well as providing the views of the Islamic Republic of Iran to international societies.

Various aspects of the ethical and legal issues in organ transplantation described in this study are shown in Table 3. In any ethical decision-making process, attention must be paid to four principles: respect for autonomy, beneficence, non-maleficence, and justice.

According to these principles, in the process of organ transplantation, information must be provided to both the donor and recipient, and the risks resulting from the transplant should be fully explained to them. The desired result must be obtained after the final decision is made by the recipient and the donor, with the utmost respect for their choices without compulsion. The profits and losses of the two parties should be evaluated and ultimately a decision is made that will have the greatest profit and the least loss for both parties.

Regarding obtaining a transplant from a living donor, obtaining informed consent, preventing material profits, assessing how to compensate for possible injuries and damage to the donor, deciding how to get organs from children and people with mental disorders, the conditions for selecting the recipient, and the ethical guidelines for the creation of organ & tissue banks are the most important ethical issues.

It seems that in cases of organ transplantation from a cadaver, scholars of the Islamic religion find that the ethical issues are more acceptable. However, this type of transplant depends on getting consent (from the deceased, assumed satisfaction, questioning relatives of the deceased regarding the removal of the organ for the transplantation).

One of the important aspects of the ethical issues related to organ transplantation is the issue of brain death and assurance of individual death. The issue of assurance of brain death is essential for the confirmation of surgery and the removal of the organ from the body of the donor: brain death must be approved by an ethics-science committee set up for this specific purpose. There must be an effort to save the individual’s life (especially an organ donation volunteer) and avoid material profits. There are two clinical conditions that are very close to the brain death state: the first is a persistent vegetative state caused either by a period of disruption in the oxygenation of the brain tissue, traumatic brain injury or stroke. The second is a newborn encephalopathy resulting in a brain without the upper surfaces, which means the patient cannot survive. Since both of these conditions are associated with very severe neurological damage and will eventually lead to death, there are discussions in different countries about the possible use of organs from a patient in one of the above states. At present, although licenses have been issued in a limited number of cases, most communities are opposed to organ transplantation under these circumstances. Of course, transplantation problems in newborns and the absence of an organ suitable for transplantation, has resulted in suggested use of organs from a newborn with encephalopathy. The suffering of the newborn with encephalopathy, the inability to treat them and, on the other hand, the mental and emotional relief of the parents of the newborn who needs the transplant are possible reasons justifying this action [54].

The removal of unimpaired vital organs from brain death and other classes of patients such as vegetative states and newborns with anencephaly can be seen as the authorized killing of people for their organs [55]. One problem with this is that once utilitarian issues are used to justify killing ventilator/dependent patients who are dying; those same issues could also be used to justify killing non-ventilator/dependent patients or patients who are not dying [10].

How to choose the recipient is one of the most important ethical issues affecting organ transplantation, which, unfortunately, has the potential to result in immoral practices [56]. Sometimes, the rewards and material benefits of organ donation and its survival rate are the only factors used to identify the recipient of the transplant. The high successful likelihood of a transplant, the transplantation lifespan, life expectancy, and quality of life of the patient receiving the transplant and the rate of return to normal life are the important conditions for selecting the recipient of the organ transplant. The identification of emergency patients needing a transplant is important in organ transplantation. Obviously, other factors such as the extent to which the individual is useful to the community or the willingness of the donor to donate to a particular person can sometimes affect the predefined conditions. However, this may lead to a situation where organ transplant priority is given to those with greater social status, those with high IQ and rich people. In that case, these patients would receive an organ transplantation regardless of the lack of organs and the many other people in need of organ transplant [54]. Also, the refusal of an organ transplant for diabetics, the elderly and people close to death, as well as people with mental disorders is certainly immoral and against social justice [57].

Solving the problem of organ and tissue deficiency and the need to pay attention to ethical standards is one of the challenges in organ transplantation, which can be solved by changing the organ donation system in the country, so that each person must make a decision whether they will be an organ donor. If a person has no desire to donate at any time during their life, they should take a “non-organ donation card”. The card at the time of death indicates that they are unwilling to donate, and, thus, the healthcare system is not required to obtain consent from relatives in critical situations that sometimes cause resistance to the donation of the organ [58]. On the other hand, increasing public education in society about obtaining consent in cases of brain death, and the use of knowledgeable experienced people to talk with relatives of the deceased so that they can encourage organ donation, as well as facilitating the process of organ transplantation by providing legal strategies and taking into account the religious beliefs of the individual are all possible appropriate solutions to the problem of organ deficiency in society.

One of the important ethical issues, especially in cases where a transplanted organ is from non-kin living persons, is the compensation of the loss incurred by the donor. In this regard, although it seems normal that the organ donor, in return for losing a part of his body, is entitled to remuneration, the case where a person has sold a body organ to another for money may seem very unpleasant in the opinion of many medical ethics experts as well as the general public. Some people are opposed to any purchasing and selling of organs, and they believe that this will make it very difficult for low-income people needing a transplant. Therefore, this will challenge the concept of justice in treatment, which is an important ethical principle. On the other hand, banning the purchase and sale of organs may be considered in contradiction to the health of the community, since needy people may sell their body organs due to financial problems.

Another dimension to the ethical issues surrounding organ transplantation is the transplantation of animal species to humans [59]. Organ transplantation from animal to human is justifiable and important for reasons such as the limited transfer of human organs, the long-term expectation of patients for receiving human organs, the irreversible damage or death due to the delay or lack of reception of organs and the unlimited availability of animal species [60,61]. However, this type of transplant has opponents who believe that the acceptance of animal transplantation can lead to the risk of transmission of an unknown animal-to-human disease and the inability to control and prevent the transmission of these diseases to others. Other expressed concerns oppose animal-to-human transplantation include the lack of acceptance of the patient by the community, mental illness in the patient with an animal organ in the body, and the decrease in the quality of life of the recipient as a result of these societal pressures and their religious beliefs. Therefore, ignorance of the moral, legal and social consequences of these types of transplants may result in irreparable harm; however, the use of animal organs may help supply more organ transplantations, thus reducing the harm to the recipient, while considering animal rights.

Transplantation in children (as either the recipient or donor) is a particularly important ethical issue in organ transplantation [62,63,64]. Aramesh et al. in Iran in 2006 examined the ethical considerations in the use of children as organ donors [65]. In this review, the authors believed that none of the ethics experts agreed with the use of children as organ donors, and it should only be allowed in certain conditions, such as the lack of access to another donor for donation and the low risk of surgery for the child. Meanwhile, the precondition of informed consent approved by the ethical authorities is also compulsory. Despite the permissibility of a transplant from children under special circumstances, there is still concern around the abuse of children for organ transplants, which is a very serious issue and requires special attention.

Among other ethical challenges in organ transplantation is the use of embryonic tissue, which has cast doubt on the process of issuing abortion permits in some societies [66,67,68]. Therefore, there is opposition to the use of that tissue type. The use of genetic engineering to replicate human stem cells is a potential alternative which results in its own unique ethical issues, including the value of the resulting being, the dignity of a human being, the manipulation in divine creation, the danger and the adverse effects of this method, slippery slopes towards the production of replicated human beings and human and organ trafficking [69,70].

## 8. The Most Important Challenges in Organ Transplantation in Iran and Their Solutions

Although organ donation in Iran has greatly improved over the past years, this improvement has not been the same in all parts of the country. For example, although public perception of organ donation from cadavers has improved in recent years, the level of family satisfaction in this area is still low in some provinces of Iran, and the organ transplantation of patients from a brain-dead donor is problematic in some provinces due to the lack of a national system. Organizing a culture of donating organs and establishing a proper national system for transplantation can ensure and facilitate access for all individuals to transplant services. In addition to the poor culture of organ donation in the community, other challenges are also associated with the low rates of organ transplantation in Iran. The lack of specialized facilities and hospitals equipped for organ transplantation are among these challenges.

Although there are many centers for kidney transplantation in Iran, there are not many well-equipped centers for other types of organ transplantation. This has made the access of patients to transplant services difficult. For example, the number of centers equipped for liver, heart and lung transplantation is very limited. Ideally, centers at least at a provincial level should be equipped to provide all the transplant services. Obviously, in order to make this happen, appropriate infrastructure will need to be created, including a comprehensive organ transplant system, sufficient funding and an expert and efficient human source. The development of these centers and cooperation, not the competition between them, can help increase the quality and quantity of transplantation in Iran. The development of transplantation to cope with the increasing need of patients for this healthcare service will require the training of adequate human resources in various fields related to it, in particular physicians, nurses, social workers, and so forth. Unfortunately, there is currently insufficient planning and infrastructure in this area. Developing transplantation capability and establishing several well-equipped centers in Iran will require the implementation of accurate planning by policy-makers and health authorities in Iran to ensure the future of organ transplantation.

At present, hospitals throughout the country do not have an official strategy to encourage families to consent to the organ donation of brain-dead relatives. In this way, the establishment of specialized counseling workshops for these families in hospitals can be helpful [71].

The lack of a national information bank on organ transplantation and donation to inform people about the statistics of brain deaths is also a challenge that makes identifying such individuals difficult for hospitals. One of the requirements for planning for the future is having sufficient and accurate information on the subject. The lack of a codified and national program for organ transplantation has resulted in no accurate information. Although different centers collect information about their performance and results, there is no centralized system with accurate and up-to-date data on this topic. Transplantation centers should be required by national laws to record their activities and their outcomes in a centralized database. This information can then be used to find problems, and thus, some measures can be taken to resolve them and improve the quality and quantity of organ transplantation services in the country.

In Iran, there are several transplant centers, but each of these centers is independent of the other, and there is no integrated, coherent system for organizing and evaluating their activities. This has led to a lack of adequate, accurate information on the transplantation and its orientation in the future, and as a result, it is difficult to assess the current problems and plan to resolve them. This also makes it difficult to improve the quantity and quality of organ transplantation in the future.

It is necessary that all transplant centers are controlled by a coherent system. This will improve transplantation and patient’s access to the transplant services. One of the reasons for the lack of a consistent transplant system in Iran is the absence of national laws in this area. The only law in this field just refers to the use of organs of brain-dead patients for transplantation. Although this is a very good step for early transplantation development, it will not be a sufficient strategy in the future. Iran needs coherent rules for its policies on organ donation and its development, the increase of the quantity and quality of the transplants and the access of patients to the services, especially the coverage of the necessary insurance.

The lack of proper care of brain-dead patients in hospitals, especially in small and unequipped hospitals in cities, is also another disadvantage that poses a challenge to keeping vital organs healthy for donation. However, this challenge is solvable with the appropriate training of the care and nursing team and by equipping the wards of brain-dead patients [40].

As previously mentioned, one of the major challenges in organ transplantation is the lack of supportive laws and appropriate insurance coverage for the donor and recipient families. Since most costs are paid by the recipient, this makes it difficult for patients who need it, and sometimes they may die due to the inability to afford care. Measures should be taken to support these patients, including focusing on changing the policies of public insurance agencies and providing financial assistance via community-based organizations.

Organ transplantation is one of the most expensive practices in the healthcare system, but it has been shown that investing in this field is much cheaper and more cost-effective for the community than treating patients with organ failure. For example, the cost of kidney transplantation and its subsequent care is much less than the cost of dialysis. In addition, the lifespan and quality of life of transplanted patients are much better than non-transplanted patients. Given the limited facilities and financial resources, policymakers in the healthcare sector need to place greater priority on organ transplantation. This is important not only in terms of transplantation, but also in post-transplant care. As an example, one of the requirements for maintaining a transplanted organ is access to drugs against transplant rejection. It is necessary to provide adequate insurance coverage for easy access to these medications.

In this paper, we have presented a brief of the challenges facing Iran in the matter of organ transplants. Planning to confront and solve these challenges will ensure the country is better able to deal with illnesses that cause organ failure in future.

## 9. Strategies for the Extension of Organ Transplantation in Iran

It seems that success in cultural affairs and advertisements related to organ transplantation and donation needs to be accompanied by policies, plans and facilities for exploiting them; in this context, it is worth mentioning the “organ donation reward” scheme. In this plan, a reward is paid out as a gift from the government to acknowledge altruistic organ donation [72,73]. This can also somewhat compensate the damage incurred by the donor, as well as preventing a financial link between the recipient and the donor. It seems that institutions involved in organ transplantation need to increase the donation reward to the extent where it can hinder the establishment of unverified financial relationships between the donor and the recipient. Furthermore, government agencies, such as the bank distribute transplanted organs or other public health institutions in the community should compensate financial losses by granting sufficient rewards, long-term insurance for the donor, and monthly payments for a specified period to donors. Obviously, if there are no solutions to this problem, there will continue to be illegal and unethical financial links between the recipients and the donors, the consequences of which, can be seen in form of organ trafficking in many countries around the world.

We predict that organizing and coordinating organ transplantation activities is possible only through a comprehensive, coordinated system with an efficient organizational structure. We predict that such a system can be used for studying and predicting, policy-making, planning, implementation, and monitoring of the educational, research and medical activities of organ transplantation. Undoubtedly, this is one of the imperatives in the purposeful, rapid development of organ transplantation in Iran. It is worth noting that, as many countries have successfully demonstrated, the creation of regional scientific poles, scientific-research parks and development centers within the country will be very helpful: as organ transplantation educational centers and institutions are established in a region close to each other, the important circles of treatment will become interconnected. On the other hand, reviewing existing laws and formulating specific supportive laws with transplantation in mind, such as laws around the necessary facilities for recruiting and employing foreign scientists to utilize their knowledge and capabilities (both Iranian and non-Iranians) in education, research, treatment and the like, will support ongoing transplantation development. Studying other successful countries in this field can provide a model for removing the barriers and deficiencies in organ transplantation in Iran.

Organ transplantation is one of the new issues in medical science. It is an important achievement and a sign of the improvement and ability of medical centers. The phenomenon of brain death and the possibility of using it in the transplantation of organs, while creating new possibilities for saving of a number of patients, has resulted in a number of ethical, cultural, and legal issues. It seems necessary to enforce laws on brain death and transplantation to solve problems associated with the high cost of keeping brain-dead patients and to encourage patients in need of organ transplant. Of course, due to the obstacles in organ transplantation, brain death, and organ donation, careful examination of the issues from a medical, ethical, legal, and jurisprudential point of view is needed. These examination efforts coupled with the proper cooperation of the medical community, institutions, organizations, and related systems, are necessary to address the barriers to implementation and lack of facilities and problems in this area. Unauthorized and illegal actions can be avoided by providing and facilitating organ transplantation from cadavers. Organ transplant organizations in Iran, especially community-based organizations, combined with governmental, public, medical, and research efforts, can promote a transplantation and donation culture to assist needy patients and produce a national effort to help patients who require transplantation. In this way, media, culturally influential people, and art can all play a valuable role and, supported with proper information, can result in a society with a level of consciousness that does not consider organ donation as a taboo against its intellectual, moral, and religious values, but rather deeply believes that “Organ donation is giving the gift of life”.

## Figures and Tables

**Table 1:** The process of successful organ transplantations in Iran.

Year	Successfully Transplanted Organ/Tissue	By
1935	Cornea	Dr. Mohammad Gholi Shams
1968	Kidney	Dr. Seyed Mohammad Sanadizadeh
1991	Bone marrow	Dr. Ardeshir Ghavamzadeh
1992	Intestine	Dr. Iraj Fazel
1993	Liver	Dr. Seyed Ali Malek Hosseini
1993	Heart	Dr. Hossein Mandegar
2000	Lung	Dr. Seyed Hassan Ahmadi
2006	Pancreas	Dr. Saman Nik Eghbalian

**Table 2:** The characteristics of patients in different situations after acute brain injury including coma, vegetative state, brain death, and related disorders [16].

Situation	Arousal	Awareness	Motor Function	Respiratory Function	Electroencephalography	18F-Fluorodeoxyglucose Positron Emission Tomography	Prognostication
Coma	Absent	Absent	No voluntary movement	Depressed variable	Major generalized slowing	40% to 50% decrease	Recovery, vegetative state or death within 2 to 4 weeks
Vegetative state	Normal	Absent	No voluntary movement	Normal	Major generalized slowing	50% to 60% decrease (associative cortex is most impaired)	Depends on etiology (traumatic, non-traumatic)
Minimally conscious state	Normal	Minimal	Minimal but reproducible voluntary movement	Normal	None specific generalized slowing	20% to 40% decrease	Better than vegetative state
Locked-in syndrome	Normal	Normal	Complete paralysis except for eye movements	Normal	Near to normal	Normal/near to normal activity	Persistent quadriplegia with prolonged survival
Brain death	Absent	Absent	Spinal reflexes	Absent	Isoelectric	No activity in brain or brainstem	Irreversible

**Table 3:** Different dimensions of the ethical issues in organ transplantation.

1	The degree of individual’s autonomy over his own organs and donation to other people for organ transplantation
2	Ethical issues related to organ transplantation from a living person to a living person or from a cadaver to a living person
3	Ethical issues related to brain death and assurance of individual death
4	Ethical issues related to obtaining consent from the organ donor
5	Ethical issues related to how to select the recipient and prioritize the recipients
6	Ethical issues related to the material and spiritual rights of the donor and to ensure the definite health of the donor
7	Solving the issue of transplanted organ and tissue deficiency with attention to and observance of its ethical issues
8	Ethical issues related to the financial relationship between the donor and the recipient and its legal controls
9	The fetus as an organ donor and ethical issues related to it
10	Sexual cells and their use in tissue transplantation and related ethical issues
11	Genetic engineering in transplantation and ethical issues
12	Animal-to-human transplantation and ethical issues related to it
13	Creating organ banks and how to process organs and the ethical issues related to it
14	Organ transplantation in children (as a recipient or donor) and ethical issues related to it
